# A New Look at the Purported Health Benefits of Commercial and Natural Clays

**DOI:** 10.3390/biom11010058

**Published:** 2021-01-05

**Authors:** Alexander Incledion, Megan Boseley, Rachael L. Moses, Ryan Moseley, Katja E. Hill, David W. Thomas, Rachel A. Adams, Tim P. Jones, Kelly A. BéruBé

**Affiliations:** 1 School of Biosciences, Cardiff University, Cardiff CF10 3AX, UK; IncledionA@cardiff.ac.uk; 2 Cardiff Institute for Tissue Engineering and Repair, Cardiff University, Cardiff CF10 3BG, UK; meganboseley@hotmail.co.uk (M.B.); MosesR@cardiff.ac.uk (R.L.M.); MoseleyR@cardiff.ac.uk (R.M.); 3 School of Dentistry, Cardiff University, Cardiff CF14 4XY, UK; HillKE1@cardiff.ac.uk (K.E.H.); ThomasDW2@cardiff.ac.uk (D.W.T.); 4 School of Sport & Health Sciences, Cardiff Metropolitan University, Cardiff CF5 2YB, UK; RAdams@cardiffmet.ac.uk; 5 School of Earth and Ocean Sciences, Cardiff University, Cardiff CF10 3AT, UK; JonesTP@cardiff.ac.uk

**Keywords:** antibiotics, antimicrobial, bacteria, clay, healing, immunogenicity, particles, pH, skin, wound

## Abstract

Clays attributed to have medicinal properties have been used since prehistoric times and are still used today as complementary medicines, which has given rise to unregulated “bioceutical” clays to treat skin conditions. Recently, clays with antibacterial characteristics have been proposed as alternatives to antibiotics, potentially overcoming modern day antibiotic resistance. Clays with suggested antibacterial properties were examined to establish their effects on common wound-infecting bacteria. Geochemical, microscopical, and toxicological characterization of clay particulates, their suspensions and filtered leachates was performed on THP-1 and HaCaT cell lines. Cytoskeletal toxicity, cell proliferation/viability (MTT assays), and migration (scratch wounds) were further evaluated. Clays were assayed for antibacterial efficacy using minimum inhibitory concentration assays. All clays possessed a mineral content with antibacterial potential; however, clay leachates contained insufficient ions to have any antibacterial effects. All clay leachates displayed toxicity towards THP-1 monocytes, while clay suspensions showed less toxicity, suggesting immunogenicity. Reduced clay cytotoxicity on HaCaTs was shown, as many leachates stimulated wound-healing responses. The “Green” clay exhibited antibacterial effects and only in suspension, which was lost upon neutralization. pH and its interaction with clay particle surface charge is more significant than previously understood to emphasize dangers of unregulated marketing and unsubstantiated bioceutical claims.

## 1. Introduction

The role of clays in medicine is well-documented, including detoxification, dermatitis, hair growth, gastro-intestinal tract, and irritable bowel syndrome, renal health, bone mass loss, and even cancer treatments. Antimicrobial resistance (AMR) is the term used when a microorganism no longer responds to antimicrobial drugs. Many bacteria, by the selective pressure of antibiotic exposure in medicine and animal husbandry, have developed resistance to multiple front line and last resort antimicrobials [1,2,3,4,5]. These so called “ESKAPE” pathogens (e.g., *Enterococcus faecium,* Methicillin-resistant *Staphylococcus aureus*, *Klebsiella pneumoniae*, *Acinetobacter baumannii*, *Pseudomonas aeruginosa,* and *Escherichia coli*) are commonly associated with increasing multi-drug resistance and virulence, and often inhabit chronic wounds, cuts or other cutaneous injuries that have not healed three months post-injury [6]. They interrupt the normal wound healing process, triggering excessive and persistent inflammation and lack of response to reparative stimuli, culminating in impaired wound closure and a failure to reinstate skin barrier function [7,8,9,10,11]. For sufferers with such wounds, the consequences can be significant, with real loss of quality of life for patients and their families.

Chronic wounds cause pain, loss of function and mobility, depression, anxiety, social isolation, embarrassment, financial burdens, prolonged hospital stays, chronic morbidity, and even death [12]. In addition to the consequences for patients, the incidence and complicated treatment of chronic wounds is a distinct drain on the National Health Service in the UK. In 2012, there were 2.2 million chronic wound cases in the UK that required management costing £5.3 billion, with 18.6 million practice nurse visits, 10.9 million community nurse visits, 7.7 million GP visits and 3.4 million outpatient visits [13].

The lack of novel antimicrobial therapies in development (for various regulatory and economic reasons) is of major concern [14] with the O’Neill report [15] predicting that by 2050, AMR could result in 10 million annual deaths worldwide. Clays have several distinctly appealing properties that could give them an advantage for AMR applications. They are abundant and are thought to act through pathways that have yet to see any resistance in bacteria [16]. They are geochemically well understood and the infrastructure for their extraction and preparation already exists, with a long history of medical use. Williams et al. [16] described the efficacy of a “French Green” clay used by the French doctor Line Brunet de Courssou to treat the common mycobacterial infection Buruli ulcers, a chronic, debilitating, necrotizing disease of the skin and soft tissue [17]. The French Green Argiletz clay (CsAg02) proved to be effective against all bacterial pathogens tested [18].

The apparent antibacterial mechanisms of certain clays are poorly understood. It has been postulated that these antibacterial effects could relate to either direct contact between the bacterial cell surface and the charged surfaces of the clay grains resulting in cell lysis. Alternatively, exposure to bioreactive cations (e.g., Al^3+^ and Fe^2+^) in aqueous solution may increase the permeability of the bacterial cell membrane; the divalent cations overwhelming iron storage proteins and oxidizing to produce reactive oxygen species [19,20,21,22].

Clays present very significant challenges when trying to undertake conventional toxicological assays. Aqueous leachates generated from the clays are straightforward to analyze; however, these do not reproduce the clinical environment where typically, water-based clay poultices are directly applied to the wound [17]. Moreover, attempts to analyze even dilute clay suspensions can be problematic given that the particles obscure key biological indicators of toxicity (e.g., cytoskeletal dysfunction, viable cell counts and microscopy), due to light scattering. Microscopic particles are well known for having large surface areas and chemically active surfaces that may interfere with viability assays and microscopic analysis due to the high adsorption capacity and optical activity of organic/inorganic particulate matter [23,24,25,26,27]. Particles interfere with classic cytotoxicity assays in a highly concentration-, particle-, and assay-specific manner but this interference may be prevented by altering assay protocols and lowering particle concentrations [28].

Of the antimicrobial clays which have already been described, many have originated in hydrothermally altered volcaniclastic environments [19], including the Eifel area of West Germany [29], Amazon Rainforest [30], and Cascade Mountain range in Douglas County, Oregon [21]. Although the source of the Green clay referred to as Argiletz (i.e., Illite or Sea Clay; [31]) and/or related clay mixtures is a proprietary secret, it likely comes from the Massif Central region of France, near its Cantal stratovolcano or Chaîne des Puys [32]. In this study, we sought to examine the geological and biological properties of a range of “bioceutical” clays to characterize their particle properties and determine their in vitro safety and antimicrobial efficacy.

## 2. Materials and Methods

### 2.1. Leachate Preparation

Leachates were prepared from six different clay samples as follows: ((1) Clay A—Redmond Healing; (2) Clay B—Kaolinite; (3) Clay C—Red Clay; (4) Clay D—Premium Nutri Clay; (5) Clay E—Green clay; and (6) Clay F—Mirador Chimaque; (Table 1). An adaptation of the British Standard (BS EN 12457 Part 2) “characterisation of waste leaching test” [33] was employed. In brief, 20 mg (instead of 100 mg) of clay was weighed out to an accuracy of ±0.1 mg and placed into a sterile 15 mL Falcon tube (with a screw cap; Fisher Scientific, Loughborough, UK). The tube was filled with double-distilled water (Cole-Parmer, St Neots, UK) up to 10 mL (instead of 1000 mL), establishing a liquid-to-solid concentration of 2 mg/mL. The capped Falcon tubes were secured into a variable speed rotatory agitator for 24 h at 7 RPM. The leachate was filtered at the time of extraction by drawing 8 mL of leachate through a 0.45 μm cellulose membrane filter (Millex™ Sterile Syringe Filters; Millipore, Watford, UK) using a 10 mL sterile syringe (Cole-Parmer, St Neots, UK). The remaining 2 mL containing the clay particle sediment was retained for use in EM, whereas the filtered 8 mL was utilized for ICP-MS (Inductively Coupled Plasma-Mass Spectroscopy) and in microbiological and wound healing assays. Leachates were autoclaved and stored in the dark at 4 °C to reduce risk of microbial interference.

### 2.2. Inductively Coupled Plasma-Mass Spectroscopy

For clay samples in solution (i.e., leachates, 1 mg/mL), ICP-MS was employed to determine the inorganic elemental composition. The leachates were acidified to 10% nitric acid and processed directly through the ICP-MS. The ICP was calibrated and checked using a certified geochemical reference material, Japanese basalt JB-3a (Fuji volcano; [34]). The leachates were analyzed using a Thermo-Elemental X-Series ICP-MS equipped with a Cetac AS-500 Auto-sampler (ThermoFisher Scientific, Newport, UK). Elements present in the solution were analyzed on mass-to-charge ratios and compared to a geological standard. This was repeated three times per leachate and the mean values and standard deviations were used to determine the results. Raw data was corrected for blanks, controls, and dilutions.

### 2.3. X-ray Diffraction

X-ray Diffraction (XRD) of bulk clay samples was performed to identify the crystalline compounds. The clay samples were packed into an aluminum holder, which was placed inside a Philips PW1710 Automated Powder Diffractometer (Philips, Amsterdam, The Netherlands) using X-rays generated by Copper (CuKα) Radiation at 35 kV and 40 mA between 2 and 70° 2θ at a scan speed of 0.04° 2θ/s. Results were interpreted using Philips PW1877 APD version 3.6 and PW1876 PC-Identify version 1.0b software (Philips, Amsterdam, The Netherlands). The semi-quantitative analysis involves the measurement of the area of the main peak for each of the phases present. For some clays, the area is not indicative of the amount present. Therefore, as was stated by Johns et al. [35], Biscaye [36] and reported in Cook et al. [37], weighting factors have to be employed. The percentage error for the semi-quantitative analysis depends on what is in the sample but at most is just a few percent, which is why it is an acceptable method. Since this is semi-quantitative, errors are not usually reported. XRD patterns are shown in the Supplementary Materials, Figure S1.

### 2.4. Electron Microscopy

Clay samples were observed using different EM imaging modalities. The Field Emission Gun Scanning Electron Microscope (FEG-SEM; MAIA3, Tescan, Brno, Czechia), provided information about the surface topography and composition of the samples. SEM stubs (aluminum; Agar Scientific, Stansted, UK) were prepared with samples of dried clay. Prior to imaging, the stubs were splutter coated (SC500 Sputter coater, Bio-Rad, Hercules CA., USA) with a gold/palladium mixture to a thickness of 20 nm. Clays were imaged at a working distance of 9.1–10.2 mm, spot size 5.0 and 10.0 kV. A range of magnifications were used to obtain images of clay to allow direct comparisons of the mineral grains in terms of their morphology, surface texture, and size distributions.

Nano-scale characterization of the clay grains was observed using a high-resolution, Transmission Electron Microscope (HR-TEM; Jeol, Ltd., Tokyo, Japan). Clay samples were suspended in molecular biology grade water, vortexed for 30 s and using a pipette, 1 μL aliquots per sample were placed onto TEM grids (copper, 300 mesh size, holey carbon film; TAAB, Aldermaston, UK) and dried at room temperature for 30 min. The clays were observed under a JEM-2100 LaB6 Transmission Electron Microscope (Jeol Ltd., Tokyo, Japan), operating at 200 kV, with a high-resolution Gatan digital camera (Gatan, Inc. Pleasanton, CA., USA) capable of a resolution of 0.02 nm.

### 2.5. Filamentous Actin Measurement

Intracellular F-actin was stained by incubation of cells with fluorescein isothiocyanate (FITC)-Phalloidin probe (1.6 × 10^−6^ M) (Molecular Probes, ThermoFisher Scientific, Newport, UK). It was prepared according to the manufacturer’s protocol by first dissolving 0.1 mg (300 units) in a vial provided by the supplier, that was reconstituted with 1.5 mL of methanol (analytical grade; Fisher Scientific, Loughborough, UK). The stock was separated into small vials and stored in the dark at −20 °C until further use. F-Actin was determined in THP-1 cells to observe the cytoskeletal remodeling effects of clays and the recruitment of peripheral blood monocytes into wounds treated with clays. As these cells differentiate into macrophages upon entering the wound space, this gives an indication of the ability of clays to stimulate the immune response, in addition to remodeling that occurs when monocytes/macrophages internalize particles and necrotic tissue.

The THP-1 human cell line (i.e., leukemic monocyte; European Collection of Authenticated Cell Cultures; Merck, Watford, UK) was sub-cultured and maintained according to the supplier’s guidelines. Cells were seeded to 4 × 10^6^ cells/mL, and aliquots of 1 mL were incubated with 1 mg/mL of clay suspension/leachate, phosphate buffered saline (PBS; Sigma-Aldrich, Gillingham, UK) for the negative control, or bacterial lipopolysaccharide (LPS; Sigma-Aldrich, Gillingham, UK; [38]) as the positive control and maintained at 37 °C in a humidified 5% CO_2_/95% air atmosphere for 1 h. All assays were performed in triplicate.

Falcon tubes were centrifuged at 250× *g* for 5 min, after which the supernatant was removed and residual liquid blotted with tissue (Kimwipes; Cole-Parmer, St Neots, UK). One hundred microliters of the fixation reagent “Medium A” (from the FIX & PERM Cell Fixation & Cell Permeabilization Kit; ThermoFisher Scientific, Newport, UK) was added to all tubes, vortexed gently and incubated at room temperature for 15 min. Cells were then washed with 2 mL of PBS buffer (Sigma-Aldrich, UK) and centrifuged at 250× *g* for 5 min. The supernatant was removed, and residual liquid blotted as before. Forty microliters of Phalloidin and 2 mL of permeabilization reagent “Medium B” (Fix and Perm Kit; ThermoFisher Scientific, Newport, UK) were mixed, and 102 μL of this mixture was added to each tube. The negative control was the exception to this, instead having 100 μL of the permeabilization reagent Medium B and 2 μL of PBS instead of phalloidin. The tubes were then incubated in the dark by placing tubes in bench cupboard for 15–20 min at room temperature.

Unbound Phalloidin was removed with 5 mL of PBS and centrifuged once again at 250× *g* for 5 min. The supernatant was then removed and the pellet re-suspended in 0.5 mL PBS. This final cell suspension was then analyzed for F-actin polymerion by measuring the probe mean fluorescence intensity (MFI) using the Bio-Rad Accuri C6 Flow Cytometer (Bio-Rad, Hercules CA., USA), operating at the FL-1 channel of fluorescence.

### 2.6. Confocal Laser Scanning Microscopy

Cell suspensions from the F-actin measurements were centrifuged at 250× *g* for 5 min. The supernatant was removed, and the pellet re-suspended in a minimal amount of PBS, in this case 100 μL, to avoid crystal formation. The cells were aliquoted (≈200 μL) onto microscope slides (Fisher Scientific, Loughborough, UK) and mounted under coverslips using 25 μL of a 1:1 mix of Vectashield Hardset™ with DAPI (4′,6-diamidino-2-phenylindole) (Vector Laboratories, Upper Hayford, UK).

Prepared slides were then visualized on a Zeiss LSM880 Airyscan Confocal Laser Scanning Microscope (Zeiss, Cambridge, UK) at the Bioimaging Research Hub at Cardiff School of Biosciences. Cells were imaged with appropriate scan parameters for sequential excitation/detection of DAPI (excitation max 358 nm/emission max 461 nm) and Alexa 488 (excitation max 495 nm; emission max 519 nm) using Zeiss Zen Black software (Zeiss, Cambridge, UK). Z-stacks of optical sections were taken through the cells via Nyquist sampling using a confocal pinhole size of 1AU. Images were presented either as single optical sections or as maximum intensity projections of the Z-stacks with an accompanying transmitted light image obtained simultaneously by differential interference contrast (DIC) microscopy.

### 2.7. Keratinocyte Proliferation and Viability

The immortalized human skin keratinocyte cell line (HaCaTs) was obtained from the German Cancer Research Centre (Heidelberg, Germany). HaCaTs were cultured in DMEM, supplemented with 1% antibiotics/antimycotics (100 U/mL penicillin G sodium, 100 μg/mL streptomycin sulphate and 0.25 μg/mL amphotericin B), 2 mM L-glutamine and 10% fetal calf serum (FCS, all purchased from ThermoFisher Scientific, Newport, UK). HaCaTs were maintained at 37 °C in a humidified 5% CO_2_/95% air atmosphere, with medium changed every 48–72 h.

To assess clay effects on proliferation and viability, HaCaTs were seeded into 96-well micro-titer plates in 10% serum-containing DMEM (100 μL) at 5 × 10^3^ cells/well for 24 h, followed by incubation in serum-free DMEM (100 μL) for a further 24 h. Serum-free media was subsequently replaced with 1% serum-containing DMEM with selected leachates at 0.05 g/L concentrations (6 wells/leachate; higher concentrations up to 10 g/L caused cell death (data not shown). Cultures were maintained at 37 °C in a humidified 5% CO_2_/95% air atmosphere, with medium changed every 48 h. HaCaT proliferation and viability were assessed at 24 h, 72 h, 120 h and 168 h, by the addition of 100 μL MTT [3-(4,5-dimethyl-2-thiazolyl)-2,5-diphenyltetrazolium bromide] (5 mg/mL, Sigma-Aldrich, Gillingham, UK; [39]). After 4 h incubation at 37 °C in a humidified 5% CO_2_/95% air atmosphere, MTT was removed from each well and replaced with 100 µl DMSO (DMSO, ≥99.7%, ThermoFisher Scientific, Newport, UK). Absorbance values were subsequently measured using a Bio-Tek Instruments Microplate Autoreader (ThermoFisher Scientific, Newport, UK), at 540 nm. All assays were performed in triplicate. Clay effects on cell proliferation and viability were expressed as percent viable cells versus untreated controls, which were arbitrarily assigned a viability of 100% [40].

### 2.8. Keratinocyte Migration and Wound Repopulation

We next assessed clay leachate effects on HaCaT migration and wound repopulation, using an in vitro scratch wound model [40]. HaCaTs were seeded into 24-well plates in 10% serum-containing DMEM (1 mL) at 7.5 × 10^4^ cells/well for 48 h, followed by incubation in serum-free DMEM for another 24 h. Serum-free DMEM was removed and scratch wounds made using sterile pipettes. Following PBS washing (×2), 1% serum-containing DMEM with selected clay suspensions and leachates at 0.05 g/L concentrations (3 wells/leachate), was added and cultures maintained at 37 °C in a humidified 5% CO_2_/95% air chamber for 48 h. HaCaT migration and wound repopulation were monitored by Time-Lapse Microscopy (Cell-IQ^®^ Automated Cell Culture and Analysis System, Chip-Man Technologies Ltd., Tampere, Finland). Digital images were taken every 30 min over 48 h and scratch wound repopulation rates quantified using Cell-IQ Analyser™ Software (CM Technologies Oy, Tampere, Finland). Data were expressed as % wound closure at 24 h and 48 h, versus wound areas at 0 h. All assays were performed in triplicate.

### 2.9. Minimum Inhibitory Concentration

A broth microdilution method was performed as described by Jorgensen et al. [41]. In brief, bacterial isolates (i.e., *Acinetobacter baumannii*, *Escherichia coli*, *Klebsiella pneumoniae*, methicillin-resistant *Staphylococcus aureus* and *Pseudomonas aeruginosa*) were freshly cultured on 5% blood agar plates (LabM Ltd., Heywood, UK). Bacteria (*n* = 3 biological replicates) were then suspended in 10 mL of Tryptone Soy Broth (Lab M Ltd., Heywood, UK) and incubated overnight at 37 °C. Each bacterial culture was then diluted in PBS to an optical density between 0.08 and 0.10 at 625 nm (OD_625_) equivalent to 0.5 McFarland Standard [42]. These cultures were further diluted ten-fold in PBS prior to use.

Wells in the 96-well microtiter plate (ThermoFisher, Newport, UK) were filled with 100 µL of Cation Adjusted Mueller–Hinton (MH) Broth (Lab M Ltd., Heywood, UK). The MH-broth was used at ×2 strength in the first column of the assay to take into account the dilution effect after addition of the clay suspension/leachate. To the first column of each plate, 100 μL of suspension at 100%, 50%, and 10% starting concentrations (i.e., 600 g/L, 300 g/L, and 60 g/L, respectively) or leachate at 100%, 50%, and 10% starting concentrations (i.e., 300, 150, and 30g/L respectively) were added. The suspension/leachate was then serially diluted (1 in 2) across the plate, transferring 100 µL of broth/clay mixture each time, to a final concentration of suspension/leachate as low as 1/2048 of the starting concentration. The prepared bacterial suspensions (5 µL) were then added to each well (bar the final no growth control column), and plates were wrapped in parafilm (Sigma-Aldrich) and incubated at 37 °C for 16–20 h. Customarily the MIC is a measure of cloudiness/optical density (indicative of bacterial growth). However, as the plates with suspensions were innately cloudy to begin with, the indicator dye resazurin (30 µL; 0.1% in distilled water, Sigma-Aldrich, Gillingham, UK) was then added to each well of the microtiter plate, including the control wells to facilitate reading. (Resazurin turns from purple to pink in the presence of respiring cells). The plates were then wrapped in parafilm and incubated at 37 °C for a further 3 h. Color changes in the wells corresponding to each effective clay concentration were then recorded with purple wells indicating no bacterial growth, while pink wells were indicative of growth.

### 2.10. Statistical Analysis

Data were expressed as mean ± standard error of the mean (SEM). HaCaT proliferation/viability and migration/wound repopulation data were analyzed by one-way ANOVA with post Tukey’s post-hoc test. Significance were considered at *p* < 0.05.

## 3. Results

### 3.1. Geochemical Characterisation

All clay types were characterized by their grain size of less than 2 μm. The exception is the kaolinite sample that has a 2–5 μm size range. The mineralogy of the clays was determined using XRD. Leachable elements were determined by generating water leachates using the British Standard method BS EN 12457-2:2002 [33], followed by measurement using ICP-MS. Grain size and morphology was established by SEM and TEM. The mineralogy of the clays is shown in Table 1, along with the dried clays Munsell Chart [43] colors. The results of the ICP-MS analysis of the leachates are shown in Table 2. Only those elements that formed greater than 1% of the total elemental content of the leachate are shown.

### 3.2. F-Actin Measurement

F-actin can be measured in activated immune cells through staining with fluorescent markers and measuring this fluorescence using FACS [44], followed by visualization using CLSM. The Mean Fluorescence Intensity (MFI) obtained for each clay leachate and suspension is shown next to their respective confocal images of immortalized monocyte-like THP-1 cells (Table 3). Mean fluorescence of cells without fluorescent markers (i.e., natural auto-fluorescence of the cells) was 1068. The negative control, (THP-1 cells stained, but not incubated with either clay leachates or suspensions) was 10,206. The positive control (THP-1 cells treated with LPS) was 1,273,184. As shown in Table 3, the MFI values of all the clay leachates were much greater than their suspension counterparts, indicating increased activation of these cells by clay leachates than their suspensions. MFI levels for the suspensions were at levels like those of the negative control, indicating a low level of immune cell activation similar to immune cells in their passive state. In contrast, MFIs for the leachates of Clays A and C exceeded those of the positive control, indicating that they activated these cells to a greater degree.

The CLSM images reveal that all the cells incubated with leachates demonstrated a high degree of activation typified by thick, green-stained F-actin caps, and projections from their cell membranes known as “pseudopodia”, features also seen in the positive controls (Table 3). The negative controls demonstrate thinner F-actin caps at the cell surface, and few, if any, pseudopodia. The clay suspensions were dominated by particulate matter, though many also show whole cells.

### 3.3. Keratinocyte Proliferation and Viability

**Figure 1:**
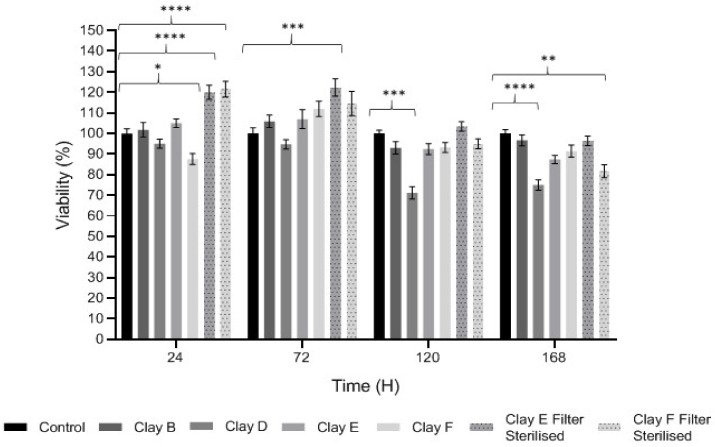
MTT analysis of HaCaT proliferation and viability, following treatment with 0.05 g/L clay suspensions or leachates (filter sterilized clays) over 168 h, versus untreated HaCaTs. Results are presented as mean ± SEM, *n* = 3 independent experiments. Significance at * *p <* 0.05, ** *p <* 0.01, *** *p <* 0.001 and **** *p <* 0.0001 versus untreated controls.

The effects of each clay suspensions and leachates on HaCaT proliferation and viability are shown in Figure 1. The MTT assay identified significant variations in the abilities of particular clay leachates to induce HaCaT cytotoxicity or stimulatory effects on HaCaT proliferation, at 0.05 g/L concentrations. Suspensions of Clay B (Kaolinite) and Clay F (Mirador Chimaque) had no significant effects on cell viability or proliferation across the 168 h time-courses (*p* > 0.05). However, Clay D (Premium Nutri Clay) suspensions exerted significant cytotoxic effects at 120 h and 168 h in culture (both *p* < 0.001). In contrast, filter-sterilized Clay E (Green) and Clay F (Mirador Chimaque) both stimulated significant HaCaT proliferation (*p* < 0.001), or close to, at early time-points of 24 and 72 h.

### 3.4. Keratinocyte Migration and Wound Repopulation

**Figure 2:**
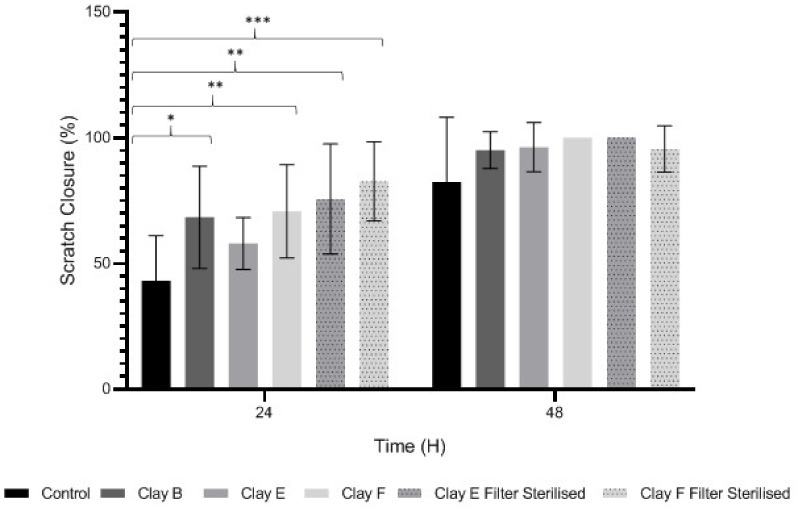
Image analysis of HaCaT migration and scratch wound repopulation, following treatment with 0.05 g/L clay suspensions or leachates (filter sterilized clays) over 48 h, versus untreated HaCaTs. Results are presented as mean ± SEM, *n* = 3 independent experiments. Significance at * *p <* 0.05, ** *p <* 0.01, and *** *p <* 0.001, versus untreated controls.

We next assessed clay suspension and leachate (0.05 g/L) effects on HaCaT migration and wound repopulation using automated in vitro scratch wounds. Time-lapse images (Table 4) and image analysis data (Figure 2) showed HaCaT migration and re-population of denuded wound spaces, with untreated controls promoting ≈80% closure over 48 h. Although wound closure was not significantly affected by clay suspensions over the 48 h culture period (*p* > 0.05, Figure 2), Clay B and Clay F suspensions and Clay E and Clay F leachates all significantly stimulated HaCaT migration and wound repopulation over 24 h period (*p <* 0.001–0.05, Table 4 and Figure 2). Despite these significant responses not being sustained up to 48 h with leachates of Clay B (*p >* 0.05) and Clay F (*p >* 0.05), enhanced HaCaT wound repopulation responses were maintained with Clay E leachate (*p <* 0.05, Table 4 and Figure 2). However, due to the significant cytotoxic effects previously identified with leachates of Clay D, this was not assessed using the in vitro scratch wound model.

### 3.5. Antibacterial Efficacy

The MIC assay is a standard method used to determine bacterial susceptibility and identifies the lowest dose at which a compound is inhibitory to the growth of bacteria [41]. All the clay leachates (see Supplementary Materials, Table S1) failed to show any effect on methicillin-resistant *Staphylococcus aureus* (MRSA) and *P. aeruginosa* at concentrations of 300 g/L.

MICs were also performed on autoclaved clay suspensions (up to double the concentration of the leachates; 600 g/L). Of the sterile clays, Clay E was the only clay to show antimicrobial activity with MICs against *P. aeruginosa*, *E. coli,* and MRSA of 75 g/L, and against *A. baumannii*, and *K. pneumoniae* of 150 g/L, indicating that they are susceptible to the Clay E suspension at these concentrations. No other clays showed any effect, regardless of their mineral composition or grain size.

The pH of the Clay E suspensions at the MIC values obtained (75 g/L and 150 g/L) was found to be in the range of 4.5–5.5 (Table 5), while the pH of wells with concentrations below 75 g/L was found to be ≥ pH 5.5. As an acid pH could confound the MIC results, all Clay E MICs were then repeated with a neutralized suspension. At pH 7, the Clay E suspension showed no antimicrobial activity, with bacteria found to grow uninhibited in all 96 wells.

## 4. Discussion

This study identified the geochemical characteristics of a selection of commercial, and supposedly bioceutical clays, and assessed them for immunogenicity and antibacterial efficacy. The geochemical characterization showed that each of the panel clays possessed mineral components with antibacterial capabilities. Clay A consisted of 72% minerals that are not considered conventional clay minerals. The morphology of the grains did not show good crystal structure, but rather morphologies usually seen in material that has been mechanically ground to a fine powder. Clay B, the Sigma “Kaolinite”, was 90% clay minerals; although, the size range of 2–5 μm is too coarse for it to be considered a conventional clay. It is likely that Clay B was derived from a kaolin deposit (e.g., China clay), formed from the chemical weathering of granite, resulting in the quartz “contamination”. EM revealed typical kaolinite platy stacked crystals. Clay C was composed of 39% clay minerals and a significant amount of non-clay minerals with 20% calcite. Similar to Clay A, the morphology suggested that the clay had been finely ground as part of the preparation. As advertised, Clay D was mostly (55%) montmorillonite, with the 8% cristobalite (i.e., high-temperature, low-pressure SiO_2_ polymorph) supporting the provenance that it comes from a volcanic area. The clay showed a range of particle sizes and some clear crystal structure. Clay E appeared to be the most bioreactive of the “clay” samples. However, it only contains 5% “clay minerals” and significantly contains 91% quartz, a mineral that has applications as the bioreactive positive control in many different types of toxicological assays [54,55]. Although good crystalline structure could be seen, the mineralogy and microscopy suggested that the clay could be more accurately described as a very finely ground quartz powder. Clay F was not commercially sourced, but was included in this analysis because of its very close geological similarity to bioreactive basaltic terrain red clays that are implicated as the causative agent in the disease “geological elephantiasis” (e.g., podoconiosis; [56]). This clay was not subjected to any grinding. The clay mineral, halloysite, has spherical cluster morphology and is a hydrated phase of kaolinite. The strong red color of the clay is attributed to the high iron oxide content.

Leachates of the clay were prepared using British Standards Institution method [33], which uses double-distilled, de-ionized water. The method is typically used in studies such as contaminated land where there are concerns about elements leaching out into groundwater. It was appropriate for this research, as it reproduces the typical clinical scenario where the dried, supposedly bioceutical clays would be mixed with water to create a poultice to apply to the skin or wound; thus, potentially releasing the constituent mineral elements into the local environment. It is important to note that the elemental levels found in the clay leachates represent a quantity that would be unlikely to harm any organism, indicating that clay leachates alone would be ineffective against bacteria. This result challenges the proposed mechanism of aluminum and iron ion action [19,20,21,22]; and supports the hypothesis that antibacterial function requires that the bacteria be directly in contact with the clay grains. Exposure to exogenous components can regulate macrophage function through their ability to affect the cell cytoskeleton by polymerization of the membrane structural protein actin (i.e., F-actin; [44]. Recruitment of peripheral blood monocytes into wounds is a key event in wound healing. Cytoskeletal remodeling occurs during extravasation of the cells through the vascular endothelium during migration from the vessel lumen into tissues. Additionally, during tissue remodeling phagocytosis of necrotic tissue by macrophages is facilitated by assembly to the cytoskeleton. The leachates of all the clays proved to be highly activating towards THP-1 immune cells; in all cases much more so than their clay suspensions, which displayed MFI readings as low as the negative controls. The low fluorescence obtained from the clay suspensions is unlikely to be due to immune cells being passive in their presence, especially given the stress directed upon them by the leachates, the constituents of which were still present in these suspensions. This could be the result of suspended particulate interference leading to reduced fluorescence transmission, or obstruction of binding of the fluorescent marker to the cells themselves [28]. This idea is supported by the lower levels of fluorescence (FITC-Phalloidin fluorophore) visible on the confocal images of the suspensions. Additionally, endotoxin in clay suspensions and leachates could have an impact on the actin polymerization in THP-1 cells. All clay samples were autoclaved, but it is unlikely that this would have removed all traces of endotoxin from them [57].

The effect of clay suspensions on THP-1 immune cells could not be confirmed, only inferred from the results obtained from the leachates. The least active leachates were those from Clays E (172,740) and F (453,096). The ICP-MS showed that these clays possessed the highest levels of iron ions. Exposure to iron oxide nanoparticles stimulates apoptosis (cell death) in macrophages [58]. This may explain the low fluorescence of the cells in these two leachates. Apoptosed cells would not produce fluorescence, creating an artificially low MFI value as cellular debris would be discounted in FACS analysis.

Epithelial integrity is maintained by keratinocytes. In healing skin wounds, keratinocytes migrate across the denuded wound regions, whereas keratinocytes adjacent to the migrating front are stimulated to proliferate [9]. However, in non-healing chronic wounds, keratinocyte proliferative and migratory responses are impaired, leading to failed re-epithelialization and closure. Thus, keratinocyte viability and induced proliferative and migratory responses are key events in normal dermal repair processes. The data presented herein suggests that most clays assessed have potentially beneficial effects on epithelial wound healing, considering the significant stimulatory proliferative and/or migratory effects induced by leachates of Clay B (Kaolinite), Clay E and filter-sterilized Clay E (Green), and filter-sterilized Clay F (Mirador Chimaque) versus untreated controls. Intriguingly, as HaCaT wound repopulation is dependent on the induction of both migratory and proliferative responses [40], the collective findings imply that these clays primarily promote keratinocyte migration, rather than cell proliferation. However, Clay D (Premium Nutri Clay) suspensions would potentially have adverse effects on epithelial wound repair, in light of the enhanced keratinocyte cytotoxic effects induced.

Based on the geochemistry data obtained, it can be suggested that the superior performance of these clays may be a result of the absence of large mineral complexes and the abundance of exchangeable, charged elements including calcium, iron, magnesium and aluminum, able to modulate cellular processes in favor of wound repair. Indeed, many reports have suggested the beneficial effects of such trace element supplementation on various stages of the dermal wound healing process, especially iron [59,60]. However, the distinct absence of elements in Clay B (Kaolinite) implies that in leachate form, there would be very little bioactivity exhibited.

None of the clay leachates showed any effect against MRSA and *P. aeruginosa* at concentrations of 300 g/L. This includes Clay E leachate, which has previously shown antimicrobial efficacy at concentrations as low as 50 g/L [61,62]. However, in those studies ICP-MS (Table 2) showed far greater leaching of ions than the present study, suggesting that leachable ions could be the cause of the putative antibacterial effect of Clay E leachate. Of all the clay suspensions, Clay E was the only one shown to be effective at inhibiting bacterial growth, and then understandably only when sterilized, giving MICs for *P. aeruginosa*, *E. coli,* and MRSA of 75 g/L, and for *A. baumannii* and *K. pneumoniae* of 150 g/L.

However, this antibacterial activity was lost upon pH neutralization. Interestingly, pH has never been attributed as the cause of any clay antibacterial effect in the literature, instead being viewed as essential for the solubility of aluminum, which along with iron is implicated in their mechanism of action [20,21,22]. The pH of normal skin ranges from 5 to 7 (Table 5) [45,46,47]. Outside of these ranges, bacteria begin to show signs of inhibition, although many of the bacteria present in this study have been shown to be able to survive at the pH range of Clay E (pH 4.5–5.5) namely: *P. aeruginosa* and *S. aureus* as low as pH 4.5 [52] and pH 4.0, [63] respectively; and *Acinetobacter* as low as pH 3.4 [50]. *E. coli* has many strains that can survive beyond its traditional limits, as shown by Otto et al. [63], with most strains tolerating the ranges between pH 5.4 and 8.2 [51] and *K. pneumoniae* showing optimum growth between pH 6 and 7.5 [51].

As the observed antibacterial effect decreased as the pH became more neutral, it is possible that the bacteria were inhibited, not necessarily by the Clay E suspension itself, but by the acidic pH (at < pH 5). Alternatively, this may not be the case, and bacteria present in this study may be able to survive at pH levels where the Clay E suspensions were effective. Neutralizing Clay E therefore granted bacteria neutral growing conditions, but also caused precipitation of the aluminum ions [64]. However, if aluminum and iron ions are responsible for the antibacterial effects, such as in Clay E, then the leachates would have been expected to be as effective as the suspensions, when in fact, they did not show any antimicrobial activity.

## 5. Conclusions

In summary, while many commercially-available bioceutical clays possess mineral constituents found in successful antibacterial clays, they do not possess any antibacterial effects. The leachates of these clays also lack ions in quantities necessary to cause any bacterial harm. Leachates were found to be highly toxic towards immune cells, while suspensions of the clays appeared to be less so. This is likely to be due to limitations in the experimental method used. The Green clay, Clay E, was the least immunogenic of all the clays, proving it has some therapeutic potential; there already being precedence for its success in Buruli ulcer treatment [17]. It was also the only clay to show efficacy at inhibiting bacterial growth across a range of bacteria at concentrations as low as 75 g/L, but only in suspension. This does not corroborate the findings of previous studies, which found both suspensions and leachates to be potent at concentrations starting at 50 g/L. Neutralization of normally acidic Clay E suspension caused a loss of antibacterial effect. Interactions between pH and clay surface charge present a possible model to explain these experimental results.

The only collective evidence supporting a therapeutic role for these clays as wound healing entities, is the significant stimulatory proliferative and/or migratory effects induced by the suspensions of Clay B (Kaolinite), suspensions/leachates Clay E (Green) and Clay F (Mirador Chimaque), albeit at lower concentrations (0.05 g/L), whilst other suspensions promoted cytotoxicity at these levels. That aside, clay suspensions on the whole are neutral or detrimental to wound healing efforts at concentrations that are far lower than those likely to be used commercially by the public.

Given the nearly ubiquitous presence of bacterial biofilms in wound infections, and their high tolerance to antibiotics, the antimicrobial potential of the Green clay would need to be tested against bacterial biofilms (not just planktonic bacteria as in the MIC assay). Minimum biofilm eradication concentration (MBEC) assays would need to be performed for Green clay before it could truly be considered as a useful antibiotic alternative. The concentrations at which the clay would be successful in inhibiting biofilm growth would inevitably be far higher than those of the MIC described in this study.

This research has demonstrated the potential of the Green clay as a novel antimicrobial with possible benefits to wound healing. It also suggests that the other commercially sourced clays are unlikely to possess any genuine antibacterial or wound healing properties.

## Figures and Tables

**Table 1:** Clay source, grain size, Munsell color, and minerology. TEM and SEM images of clays used in study showing their crystalline structure with details of their sources, grain size, color, and mineralogy. EM images show larger than average particles to better show the morphology.

Source, Grain Size, and Munsell Color	TEM	SEM	Mineralogy
**Clay A**	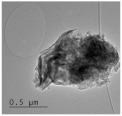	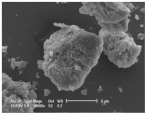	40% Cristobalite 20% Montmorillonite 16% Calcite 8% Illite 5% Quartz
“Redmond Healing” clay purchased online. <200 nm Munsell 10R 7/1			
**Clay B**	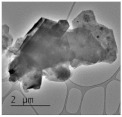	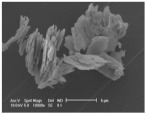	87% Kaolinite 10% Quartz 3% Illite
Kaolinite sourced from Sigma Aldrich (CAS 1318-74-7). 2–5 µm Munsell 2.5YR 8/1			
**Clay C**	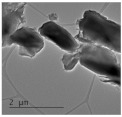	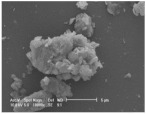	32% Montmorillonite 25.5% Quartz 20% Calcite 16% Dolomite 6.5% Kaolinite
“Red Clay” from “The Clay Cure Company”. 500 nm Munsell 2.5YR 5/6			
**Clay D**	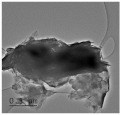	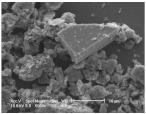	55% Montmorillonite 31% Anorthoclase 8% Cristobalite 6% Calcite
“Premium Nutri Clay” from the “The Clay Cure Company”. <200 nm Munsell 5YR 7/1			
**Clay E**	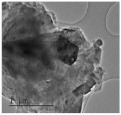	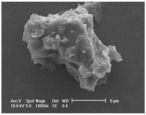	91% Quartz 3% Gypsum 3% Kaolinite 2% Illite-Smectite
Green from the Argiletz company. 500 nm Munsell 5Y 6/3			
**Clay F**	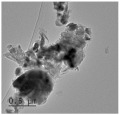	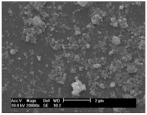	37% Kaolinite (halloysite) 23% Hematite 17% Magnetite 17% Illite 6% Anorthoclase
From Mirador Chimaque, Tenerife. <200 nm Munsell 2.5YR 4/6			

**Table 2:** Major component elements of the clay leachates as determined by ICP-MS. Values shown (mg/L) with percentage of the total elemental content for the leachate shown below.

Clay	Na (mg/L)	Mg (mg/L)	Al (mg/L)	P (mg/L)	K (mg/L)	Ca (mg/L)	Mn (mg/L)	Fe (mg/L)	Total (mg/L)
**A**	31.50	0.30	0.33	0.00	0.68	0.43	0.01	0.10	33.39
	94.32%	0.89%	0.98%	0.00%	2.05%	1.30%	0.03%	0.30%	99.86%
**B**	0.13	0.07	0.03	0.05	0.20	0.01	0.00	0.00	0.51
	25.90%	14.74%	5.55%	10.17%	40.20%	1.96%	0.30%	0.25%	99.08%
**C**	0.57	2.21	0.23	0.02	1.01	1.49	0.00	0.12	5.68
	10.05%	38.85%	4.12%	0.34%	17.77%	26.23%	0.01%	2.03%	99.39%
**D**	3.17	1.00	0.27	0.02	1.43	1.46	0.01	0.19	7.56
	41.98%	13.25%	3.54%	0.24%	18.92%	19.30%	0.07%	2.53%	99.82%
**E**	0.03	0.90	0.68	0.01	0.03	2.87	0.21	1.65	6.48
	0.51%	13.92%	10.45%	0.15%	0.43%	44.35%	3.22%	25.41%	98.43%
**F**	0.41	0.11	0.54	0.01	0.49	0.00	0.02	0.36	1.93
	21.22%	5.54%	27.75%	0.75%	25.14%	0.00%	0.94%	18.76%	99.49%

**Table 3:** FACS analysis and CSLM imaging (100×) of Alexa Fluor 488^®^ Phalloidin stained THP-1 cells following exposure to suspensions or leachates of clays A-F. MFI indicates mean fluorescence intensity, a measure of the amount of fluorescently tagged F-actin found in THP-1 cells. The negative control shows inactivated cells with the MFI obtained reflective of their inactive state. The positive control shows cells that are highly activated by LPS with correspondingly significantly higher MFIs.

Clay	Leachates	MFI	Suspensions	MFI
**A**	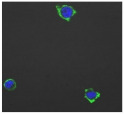	1,227,394	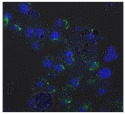	3519
**B**	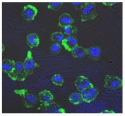	640,023	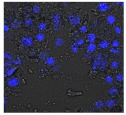	397
**C**	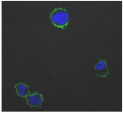	911,285	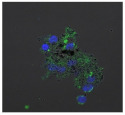	40,541
**D**	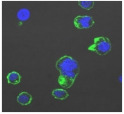	535,868	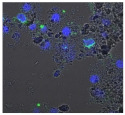	5441
**E**	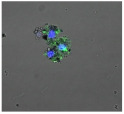	172,740	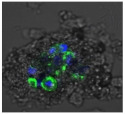	21,127
**F**	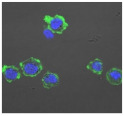	453,096	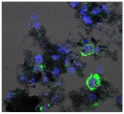	71,004
	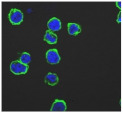	10,206 (Negative control)	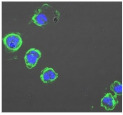	1,273,184 (Positive control)

**Table 4:** Representative time-lapse microscopy images of HaCaT migration and scratch wound repopulation, following treatment with 0.05 g/L clay suspensions or filter sterilized leachates over 48 h, versus untreated HaCaTs. Results are presented as *n* = 3 independent experiments. White lines show the mean % wound closure for the control condition at each timepoint. Scale bar = 200 μm.

Clay	0 h	24 h	48 h
**Control**	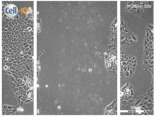	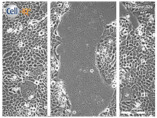	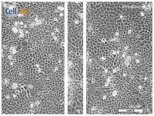
Untreated			
**Clay B**	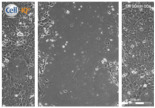	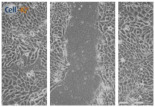	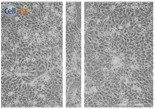
A 0.05 g/L Kaolinite clay suspension			
**Clay E**	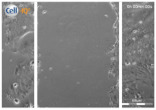	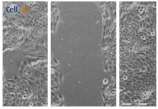	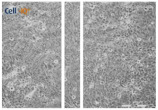
A 0.05 g/L “Green” clay suspension			
**Clay F**	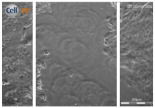	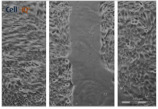	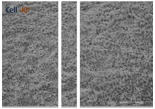
A 0.05 g/L “Mirador Chimaque, Tenerife” clay suspension			
**Clay E**	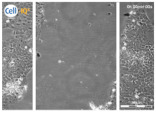	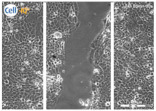	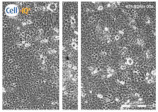
Filter Sterilized Filtered from a “Green” clay suspension.			
**Clay F**	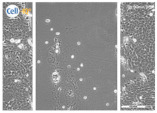	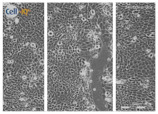	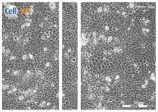
Filter Sterilized Filtered from a “Mirador Chimaque, Tenerife” clay suspension.			

**Table 5:** pH ranges at which skin, chronic wounds and the bacteria used in this study can grow. Vertical lines indicate the area in which antibiotic effect was noted. Effects at ≤ pH 4.5 were not assessed in this study. MRSA, methicillin-resistant *Staphylococcus aureus.*

pH	3		4		5		6		7		8		9		10		Reference
Normal Skin																	[45,46,47]
Chronic Wounds																	[48]
*A. baumannii*																	[49,50]
*E. coli*																	[51]
*K. pneumoniae*																	[51]
*P. aeruginosa*																	[52]
MRSA																	[53]

## Supplementary Materials

The following is available online at https://www.mdpi.com/2218-273X/11/1/58/s1: Figure S1: XRD Patterns; Table S1: Minimum Inhibitory Concentration.
