# Supplementary material for: A New Look at the Purported Health Benefits of Commercial and Natural Clays
Source: Biomolecules. 2021 Jan 5;11(1):58. doi: 10.3390/biom11010058 (PMC7824833; doi:10.3390/biom11010058)

Clay A, analyses 1 and 2: 40% Cristobalite, 20% Montmorillonite, 16% Calcite, 8% Illite, 5% Quartz

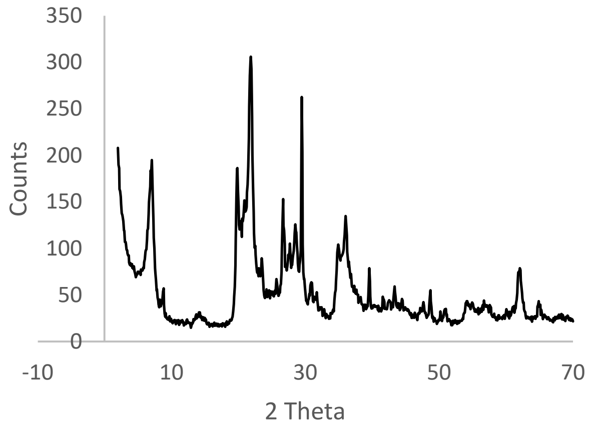

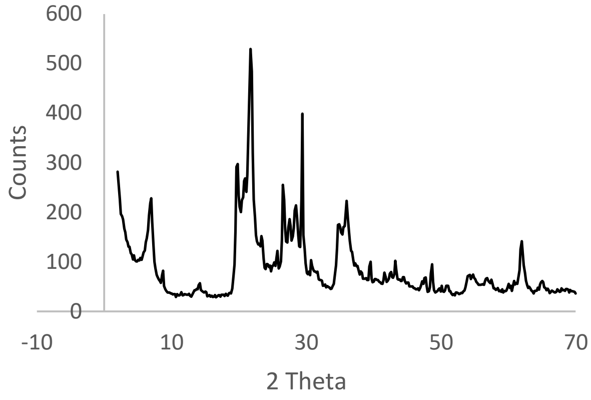

Clay B, analyses 1 and 2: 87% Kaolinite, 10% Quartz, 3% Illite

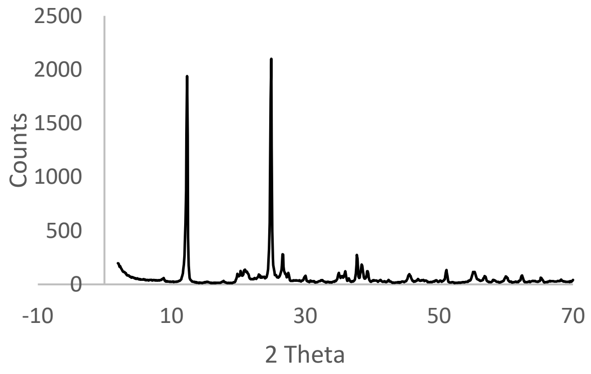

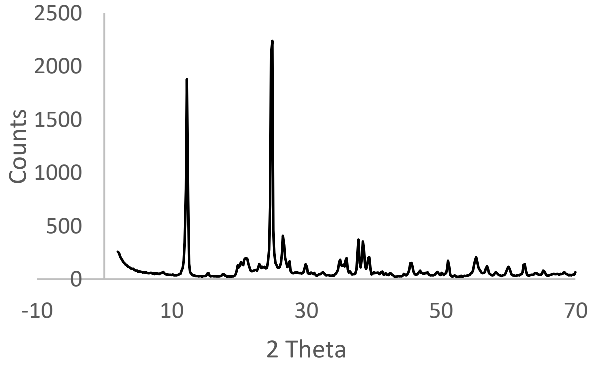

Clay C, analyses 1 and 2: 32% Montmorillonite, 25.5% Quartz, 20% Calcite, 16% Dolomite, 6.5% Kaolinite

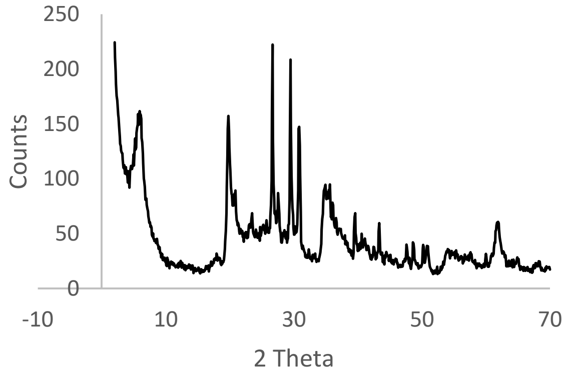

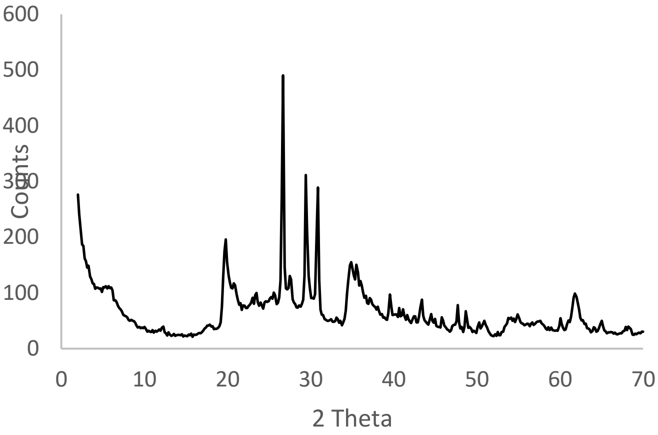

Clay D, analyses 1 and 2: 55% Montmorillonite, 31% Anorthoclase, 8% Cristobalite, 6% Calcite

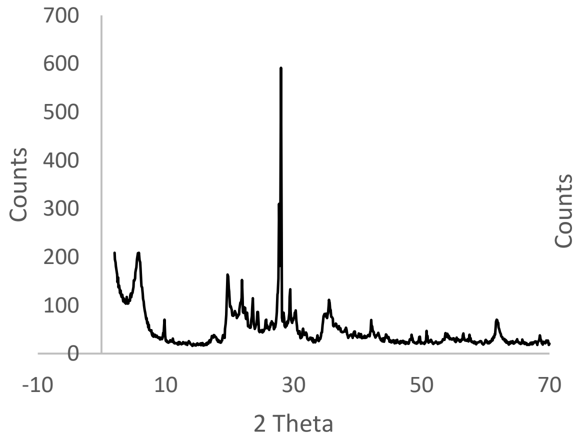

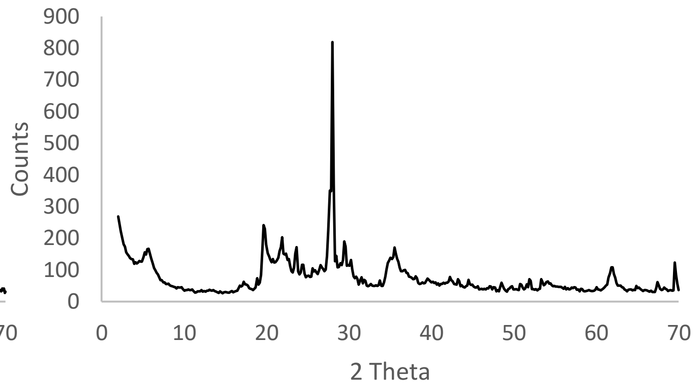

Clay E, analyses 1 and 2: 91% Quartz, 3% Gypsum, 3% Kaolinite, 2% Illite-Smectite

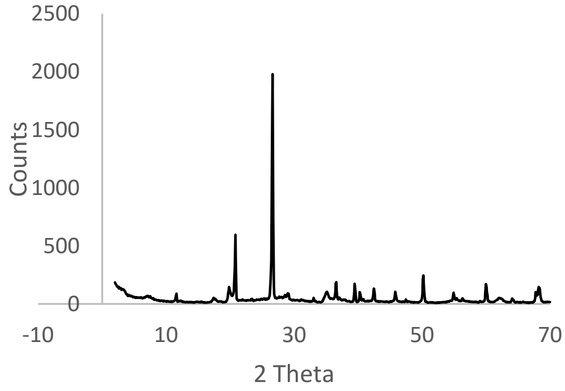

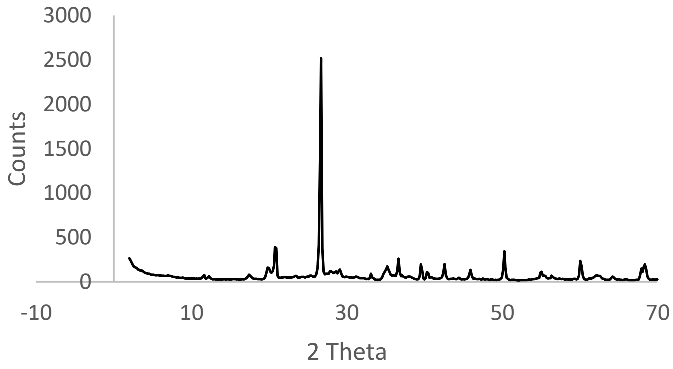

Clay F, analyses 1 and 2: 37% Kaolinite (halloysite), 23% Hematite, 17% Magnetite, 17% Illite, 6% Anorthoclase

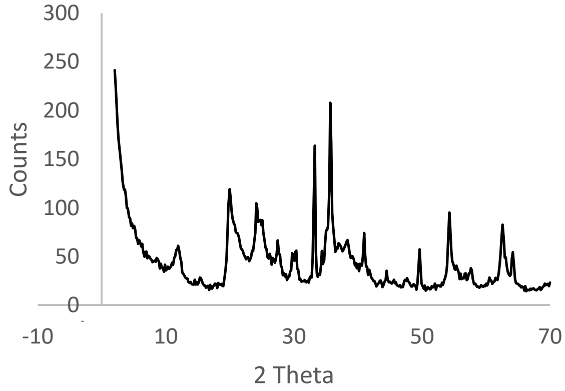

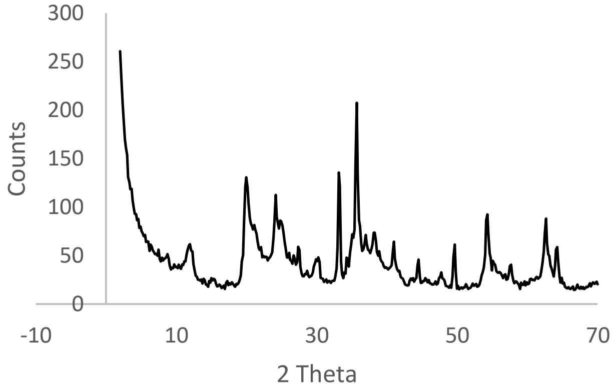

Supplement: Supplementary file 1 [file biomolecules-11-00058-s001.zip › Suppl materials/Figure S1 XRD Patterns.pdf]
