# Supplementary material for: A New Look at the Purported Health Benefits of Commercial and Natural Clays
Source: Biomolecules. 2021 Jan 5;11(1):58. doi: 10.3390/biom11010058 (PMC7824833; doi:10.3390/biom11010058)
Supplement: Supplementary file 1 [file biomolecules-11-00058-s001.zip › Suppl materials/Table S1 MIC.pdf]

Supplementary Table 1. Minimum Inhibitory Concentration (MIC) for Clays A-F.

Clay	State	Minimum Inhibitory Concentration				
		**MRSA (g/L)**	***P.aeruginosa* (g/L)**	***E. coli* (g/L)**	***K. pneumonia* (g/L)**	***A. baumannii* (g/L)**
**Clay A**	L	>300	>300	-	-	-
	S	>600	>600	>600	>600	>600
**Clay B**	L	>300	>300	-	-	-
	S	>600	>600	>600	>600	>600
**Clay C**	L	>300	>300	-	-	-
	S	>600	>600	>600	>600	>600
**Clay D**	L	>300	>300	-	-	-
	S	>600	>600	>600	>600	>600
**Clay E**	L	>300	>300	-	-	-
	S	75	75	75	150	150
**Clay F**	L	>300	>300	-	-	-
	S	>600	>600	>600	>600	>600
